# Eosinophilic Esophagitis Is an Underlying Cause for Gastrointestinal Concerns in Children

**DOI:** 10.3389/fped.2018.00113

**Published:** 2018-05-03

**Authors:** Kunsong Lee, Glenn T. Furuta, Nathalie Nguyen

**Affiliations:** ^1^Gastrointestinal Eosinophilic Diseases Program, Department of Pediatrics, University of Colorado School of Medicine, Aurora, CO, United States; ^2^Department of Pediatrics, Dankook University College of Medicine, Cheonan, South Korea; ^3^Section of Pediatric Gastroenterology, Hepatology and Nutrition, Digestive Health Institute, Children's Hospital Colorado, Aurora, CO, United States

**Keywords:** include eosinophilic oesophagitis, dysphagia, feeding disorder, pediatric, children

## Abstract

Eosinophilic esophagitis (EoE) is a chronic immune antigen-mediated disorder characterized by symptoms of esophageal dysfunction in combination with dense esophageal eosinophilia. The clinical presentation of EoE can vary depending on children's age and their ability to report symptoms, therefore a high index of suspicion for EoE is required because children and teenagers may develop coping strategies around eating. The development of symptoms measurement tools in EoE assists in not only assessing symptoms, but also coping strategies children may have developed. While the diagnosis of EoE requires endoscopic evaluation with histologic assessment of esophageal mucosal biopsy samples, several emerging methods to assess and survey the esophageal mucosa have been developed. Advances in the field to better understand the natural history, clinical and molecular features of phenotypes in EoE will be important in considering novel therapeutic options and assessing outcomes.

## Background

Since the advent of flexible endoscopy in the 1960's, gastroesophageal reflux disease (GERD) was identified as the most common cause of gross and histological evidence of esophagitis. During the last 2 decades, an emerging body of clinical experiences and research studies have identified eosinophilic esophagitis (EoE) as the next most common cause of esophagitis. The incidence of EoE ranges from 5 to 10 cases per 1,000,000 [1] and it has been reported to occur worldwide [1–3].

EoE is a chronic immune antigen-mediated disorder characterized by dense esophageal eosinophilia with symptoms of esophageal dysfunction [4]. Other causes of esophageal eosinophilia include infectious esophagitis (such as herpes simplex virus or candida esophagitis), Crohn's disease, collagen vascular diseases, drug- associated esophagitis or hypereosinophilic syndrome, but are far less common [4].

### Demographic features and association with other diseases

Although EoE was initially reported in adults, much of the early literature was described EoE in children [5]. Males are 3–4 times more commonly to be affected than females and Caucasians are more likely to be affected than other races [5]. Genomewide association studies and twin concordance studies suggest a genetic predisposition to EoE [6–8]. Genetic variations that are associated with EoE include thymic stromal lymphopoietin (TSLP), filaggrin, eotaxin-3, and calpain-14 [6, 7, 9–11]. In patients with connective tissue disorders, such as Marfan's syndrome, Ehlers-Danlos syndrome, and joint hypermobility syndrome, the prevalence of EoE is increased 8-fold, further suggesting genetic variants play a role in EoE [12].

In addition to genetic predisposition, environmental factors also play a role. Early life exposures, such as antibiotics during infancy, increases the probability of developing EoE [13]. The majority of patients with EoE have concomitant atopic disorders such as asthma, eczema, allergic rhinitis, and food allergies [14]. The prevalence rate of atopic disorders in EoE patients is three times higher than in the general population [15]. Atopic diseases that occur more frequently in patients with EoE include allergic rhinitis, asthma, eczema, and IgE mediated food allergies [16, 17].

### Clinical symptoms

The most common symptoms associated with EoE in children include feeding problems, abdominal pain and vomiting [4, 18–20]. The clinical manifestations of EoE in children can vary depending on children's age and their ability to report symptoms (Table 1) [14, 18, 21]. For instance, infants and young children can present with vague symptoms including feeding difficulties that lead to prolonged mealtimes, food refusal, gagging or GERD-like symptoms such as heartburn, regurgitation or vomiting [4, 18]. In these circumstances, a high index of suspicion is needed for EoE since oftentimes patients have developed coping strategies to ensure that food products can continue to be consumed. Children may not exhibit gagging but may use copious amounts of water or liberally use ketchup, butter or sauces to help lubricate their food or chew food to the point it is a slurry consistency [22]. Some children, learn to chew slowly, or avoid certain foods that they have difficulty swallowing such as rice, breads or meats [22]. Teenagers and adults may develop similar behaviors but most often present with a stereotypical history of prolonged dysphagia or repeated near or complete esophageal food impaction (Table 1) [18, 19].

**Table 1:** Clinical manifestations of EoE in children and teenagers.

**Younger children**	**Teenagers**
Feeding difficulties	Dysphagia
Gagging Nausea	Food impaction Chest Pain
Food refusal	Vomiting
Regurgitation	Regurgitation
Vomiting	
Food or foreign body impaction	
Failure to thrive	
Abdominal pain	

### Symptom measurement tools

Patient reported outcome tools have been developed to assess common symptoms of EoE and compensatory behaviors seen in EoE. In pediatrics, the Pediatric Eosinophilic Esophagitis Symptom Score (PEESS) uses both a child and parent score to assess symptoms associated with EoE [23, 24]. The PEESS v 2.0 consists of a module for parents and a module for children and teens (age 8–18) [23]. PEESS v2.0 has shown to correlate with specific parent reported symptoms and markers of esophageal inflammation [24]. In adults, symptom scoring tools include Eosinophilic Esophagitis Symptom Activity Index (EEsAI) and the Dysphagia Symptom Questionnaire (DSQ) [25, 26].

### Clinical phenotypes

Increasing clinical experiences and research studies suggest that a number of different clinically relevant phenotypes may exist. For instance, some patients and family members may be more prone to develop esophageal strictures whereas others do not. Additionally, some patients may respond to dietary treatment whereas others continue to have symptoms and inflammation despite limiting specific foods. Whether these observations relate to the lack of knowledge related to the natural history or the inability to perform adequate testing for food allergens is not yet known, but the clinical characterization of these groups may be helpful in understanding treatment practices and pathophysiological mechanisms. Patients with more of a fibrostenotic phenotype [27, 28] may present with food impactions or severe dysphagia, have endoscopic findings suggestive of esophageal narrowing or esophageal stricture [27, 28], and have histologic evidence of lamina propria fibrosis. Identification of this group of patients permits not only immediate attention to reducing inflammation with the hope of preventing future food bolus impactions and esophageal stricture formation. Future studies determining key therapeutic targets in this group may allow for earlier detection and novel treatment approaches.

Another phenotype are patients with EoE type symptoms, dense esophageal eosinophilia, who respond both clinically and histologically to high doses of proton pump inhibitors (PPIs) [29]. This group of patients has been thought to have a condition called PPI-responsive esophageal eosinophilia (PPI-REE) [30–32]. Interestingly, upwards of 50% of children and adults with dense esophageal eosinophilia may respond to high doses of PPIs [29, 33]. *In vitro* modeling suggests that PPIs may have mechanistic properties outside of its acid abolishing role and in fact can act by transcriptionally down regulating key eosinophil related cytokines such as eotaxin-3 [30–32]. Several studies have found no difference between the demographics, clinical presentation, endoscopic and histologic findings in PPI-REE and EoE [34, 35]. These studies suggest that subjects with PPI-REE, in most circumstances, should be considered a phenotype of EoE [29]. Advances in the field to better understand differences and similarities in the clinical and molecular features of these phenotypes will be critical to consider with respect to therapeutic options and assessing outcomes.

## Diagnostic evaluation

The diagnosis of EoE is made when a patient exhibits symptoms consistent with esophageal dysfunction, is found to have dense esophageal eosinophilia and other causes, in particular GERD, have been ruled out. A number of other tests may be helpful in further characterizing patients as listed below.

### Radiologic evaluation

**Figure 1:**
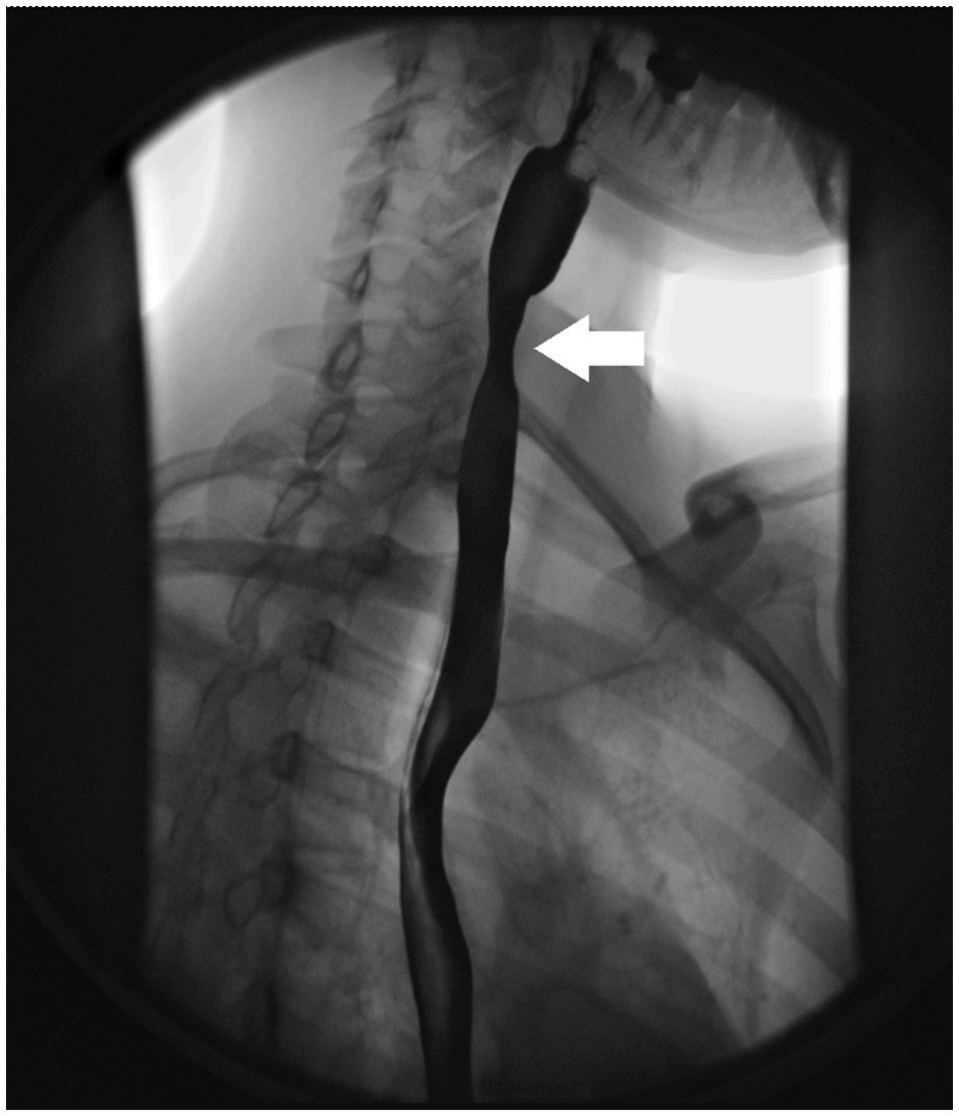
Radiologic findings in EoE. Focal esophageal stricture in the upper esophagus (white arrow).

Radiologic imaging patterns associated with EoE include focal esophageal strictures (Figure 1), ring-like indentations or subtle long segment luminal narrowing (small caliber esophagus) [36–38]. A esophagram with a barium coated pill can be used to detect these findings in patients with symptoms suggestive of EoE. In pediatric patients with EoE, an esophagram may be a more sensitive marker for esophageal narrowing than endoscopy [39]. In a study of 22 pediatric subjects who underwent both esophagram and endoscopic assessment, an esophageal stricture was identified by esophagram, but not by endoscopy in 55% of subjects [39]. In a recent study in 70 adults comparing endoscopy to radiographic imaging in adults, strictures were identified in 58% of patients by fluoroscopic study, compared to 40% on endoscopy [40]. Radiologic imaging may be more sensitive than endoscopy for identifying strictures due to the subtle long segment or diffuse narrowing that is seen in EoE, rather than focal strictures, which are more easily identifiable during endoscopy. Radiologic imaging complements endoscopy in the identification of esophageal strictures and aids in directing the management of esophageal strictures at the time of endoscopy. If an esophageal narrowing is identified on radiologic imaging, the diagnosis of EoE should be considered.

### Endoscopic evaluation

**Figure 2:**
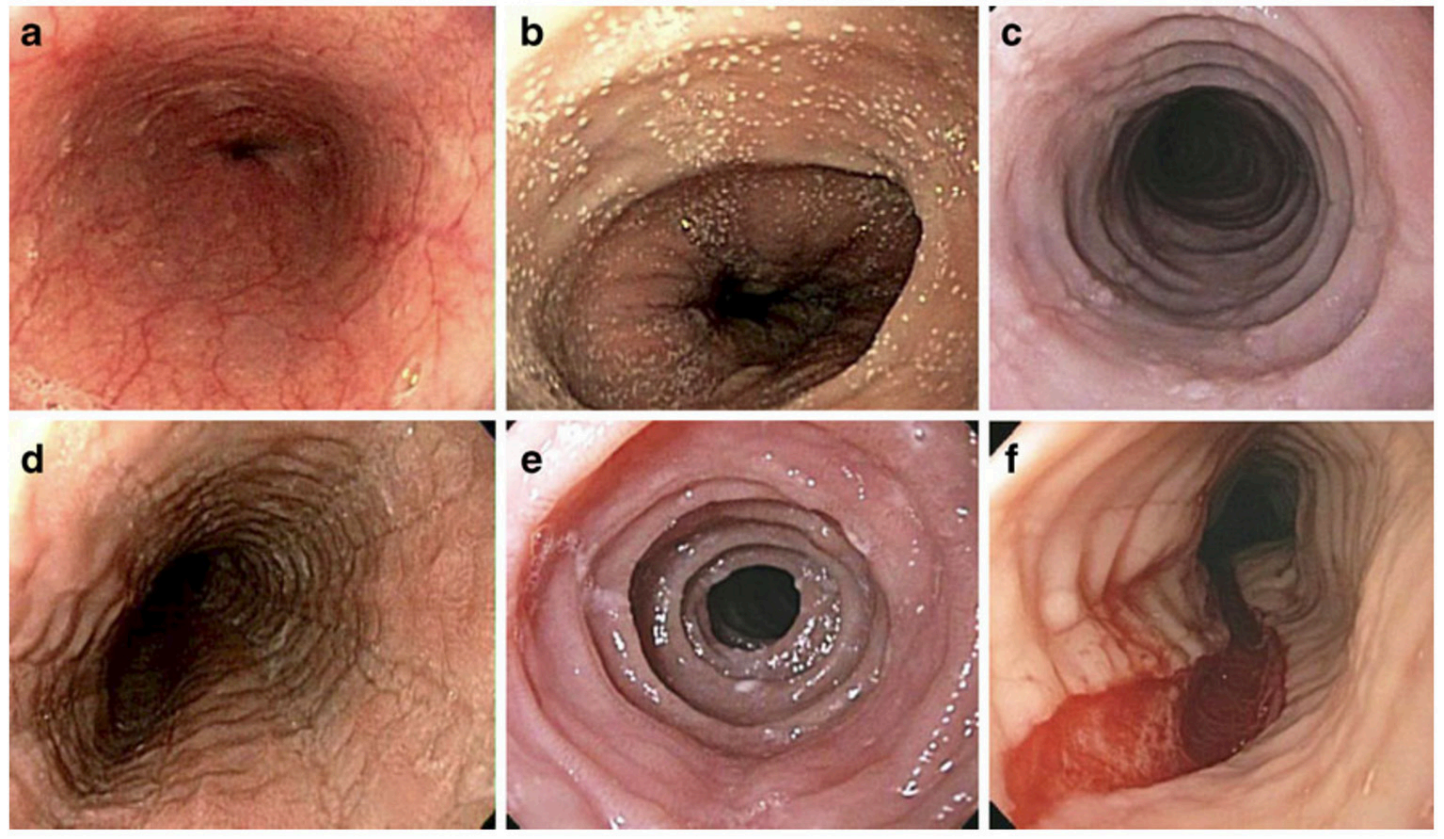
Endoscopic Features of EoE. Endoscopic features of EoE that demonstrate **(a)** normal esophageal mucosa with vascular pattern and smooth surface, **(b)** white pinpoint exudate, **(c)** concentric rings and linear furrows, **(d)** linear furrows, **(e)** concentric rings, **(f)** longitudinal tear. (Adapted from [41], with permission.

Several endoscopic findings are associated with EoE including esophageal edema (decreased vascularity), esophageal rings (or trachealization), white exudate (eosinophilic pus), longitudinal furrows, esophageal strictures, narrow caliber esophagus, and crepe paper esophagus (mucosal fragility) (Figure 2). The Endoscopic Reference Score (EREFS) is a scoring system that grades the presence and severity of endoscopic features including Edema, Rings, Exudate, Furrows and Stricture with a numerical score [42]. A recent study of pediatric subjects concluded that the EREFS score accurately identified children with EoE and response to treatment [43]. The EREFS score provides a standard method of assessing the endoscopic appearance of EoE.

### Histological evaluation

**Figure 3:**
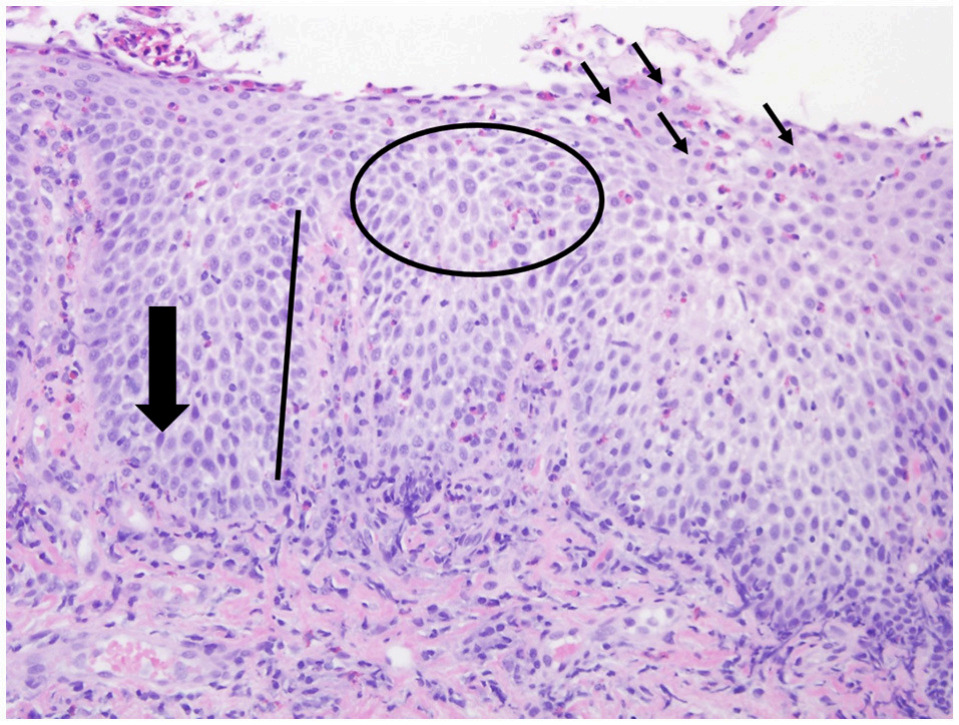
Histologic features of EoE. Histologic features associated with EoE including esophageal eosinophilia (small black arrows), basal cell hyperplasia (large black arrow), dilated intercellular spaces (circle), rete-peg elongation (black line).

The normal esophagus is devoid of eosinophils. The current gold standard for diagnosis of eosinophilic esophagitis is a histologic assessment endoscopically obtained from mucosal biopsy samples with eosinophil predominant inflammation of the esophageal epithelium (cut off value of >15 eosinophils per high power field) [4]. During endoscopy, 2–4 biopsies should be obtained from both the proximal and distal esophagus [4]. Several other histologic features have been associated with eosinophilic esophagitis including basal cell hyperplasia, dilated intercellular spaces, rete-peg elongation, and lamina propria fibrosis (Figure 3) [44, 45]. Additionally, eosinophilic microabscesses and eosinophil layering of the surface epithelium can be seen [45].

### Emerging methods of evaluating EoE

Currently, diagnosis and monitoring of disease activity is done by assessing the esophageal mucosa and by histopathological assessment of biopsies obtained during an esophagogastroduodenoscopy (EGD), which can be time consuming, expensive, and has risks associated with anesthesia. Recently, unsedated transnasal esophagoscopy (TNE) has been performed successfully in a small cohort of patients as an alternative to EGD for EoE surveillance. Unsedated TNE has advantages because it can be performed in an outpatient clinic room and requires no anesthesia or sedation [46]. Endomicroscopy methods, such as reflectance confocal microscopy, may allow the evaluation of the entire length of the esophagus with assessment of eosinophil counts and other microscopic features associated with EoE without procurement of biopsy samples [47]. New and less invasive methods to assess inflammation in the esophageal mucosal surface have also been developed including the esophageal string test, Cytosponge, and esophageal brushings [48–51]. These techniques are based on obtaining esophageal luminal effluents in order to monitor disease activity in a less invasive method than endoscopy. pH impedance monitoring has shown that subjects with EoE have lower baseline impedance values, a marker of mucosal integrity. Therefore, this tool may play a role in assessing mucosal integrity with treatment in EoE [52–54]. Additionally, the use of endoluminal functional lumen imaging probe (EndoFLIP) provides a functional assessment of the esophagus in EoE. EndoFLIP is an endoscopic catheter-based tool that determines esophageal distensibility as a measure of compliance [55–57].

## Treatment

The goals of treatment in EoE include relieving symptoms and histologic improvement of esophageal inflammation. As a result, follow up of patients with EoE is key to assess clinical symptoms and endoscopic and histologic findings. Treatment options in EoE include diet therapy, drugs, and dilation.

### Diet therapy

In 1995, a study of 10 children with esophageal eosinophilia were treated with an amino-acid based formula and all demonstrated symptomatic and histologic improvement with rebound when foods were added back [58]. Since then, a number of other studies have found that elemental diets are effective in 91% of patients, suggesting an immunologic mechanism to intact dietary proteins in the pathogenesis of EoE [59–62]. Although highly effective, concerns regarding compliance to therapy exist due to the palatability and effect on quality of life.

Empiric elimination diets are also used in the treatment for EoE. This approach does not rely on food-allergy testing, but involves the empiric elimination of the six most allergenic foods (milk, wheat, soy, eggs, peanuts/tree nuts, and fish/ shellfish). The six-food elimination diet (SFED) has shown clinical and histologic improvement in 74% of children [63]. Other studies have shown histologic improvement ranging from 50 to 81% [62, 64–66]. The four-food elimination diet consisting of the elimination of milk, wheat, egg, and soy, has also recently shown clinicopathologic improvement in 54% of patients [67]. The use of a step-up strategy for empiric elimination diets has been recently described, where patients start with empirically eliminating the 2 most common food triggers in EoE (milk and wheat) and step up to the four-food elimination diet and subsequently to the six-food elimination diet if they do not respond [68]. This strategy allows earlier identification of food triggers when using an empiric elimination diet [68].

Allergy testing directed eliminations diets involves the use of use of skin-prick, atopy-patch, or specific serum IgE testing by an allergist to determine which foods to eliminate from the diet. Directed elimination diets have had variable results. A meta-analysis showed that the overall efficacy of allergy testing directed elimination diets was 46% [62].

### Drugs

#### Proton pump inhibitors

Part of the original consensus guidelines for the definition of EoE published in 2007 recommended a trial of proton pump inhibitor (PPI) or normal pH probe study to rule out GERD as a cause of esophageal eosinophilia [4]. However, updated consensus recommendations in 2011 described a novel phenotype, PPI- REE, referring to patients with symptoms of EoE who have clinical and histological improvement on PPI therapy alone [69]. A recent meta-analysis showed that in patients with EoE, PPIs achieve histological remission in over 50% of patients [70]. Other studies found that subjects with PPI-REE have similar clinical, endoscopic, histologic, and molecular features with overlap in Th2 immune mediated inflammation and gene expression [29]. *In vitro* studies also suggest that PPIs may have an anti-inflammatory effect that is independent of the ability to block acid [30–32]. Thus, PPIs use in patients with EoE may help with concomitant/co-morbid GERD or for potential anti-eosinophil effects.

#### Topical corticosteroids

Swallowed topical corticosteroids are the only pharmacologic treatment for EoE. Topical corticosteroids can be administered to the esophagus in a swallowed form from a metered dose inhaler such as fluticasone or as an oral viscous preparation of budesonide. When using the metered dose inhaler, patients are taught to swallow after the spray from the inhaler. Oral viscous budesonide combines liquid budesonide that is intended for a nebulizer with a substrate, typically Sucralose (Splenda) in order to make a slurry [71, 72]. Efficacy of topical corticosteroids ranges from 60 to 90% [72–74]. Oral viscous budesonide has been found to be more effective, which suggests that esophageal mucosal contact time of topical corticosteroid may play an important role [75]. Potential side effects of topical corticosteroids include thrush or *Candida* esophagitis, adrenal insufficiency, or bone demineralization.

#### Esophageal dilation

Esophageal dilation is used as a treatment in EoE in patients with focal esophageal strictures or long segment esophageal narrowing. Esophageal dilation does not treat the underlying eosinophilic inflammation, and for that reason is recommended specifically for patients with EoE with esophageal strictures. The most common complication post endoscopic esophageal dilation is chest pain where as other complications such as perforation or hemorrhage are rare [76–78].

## Summary

The clinical presentation of EoE can vary depending on children's age and their ability to report symptoms, therefore a high index of suspicion for EoE is required because children and teenagers may develop coping strategies around eating. Symptom measurement tools have been developed, such as the PEESS in pediatrics, to assess common symptoms and compensatory behaviors seen in EoE. The diagnosis of EoE requires upper intestinal endoscopy for histologic assessment with enumeration of eosinophils, however a number of emerging methods to find less invasive ways to assess and survey the esophageal mucosa have been developed and used in EoE including transnasal esophagoscopy, the Esophageal String Test, cytosponge, esophageal brushings, EndoFLIP, pH impedance probe monitoring, and confocal microscopy. Treatment in EoE includes drugs, dietary elimination, and esophageal dilation. Future studies and advances in the field to better understand the natural history, clinical and molecular features of different phenotypes in EoE will be key in considering novel therapeutic options and assessing outcomes.

## Author contributions

NN, KL, and GF: all contributed to the concept development, writing and review of this manuscript and provided final approval of the version to be published.

### Conflict of interest statement

GF Co-Founder for EnteroTrack, Consultant to Shire, Royalties from UpToDate. The other authors declare that the research was conducted in the absence of any commercial or financial relationships that could be construed as a potential conflict of interest.
